# Platelet Concentrates in Musculoskeletal Medicine

**DOI:** 10.3390/ijms21041328

**Published:** 2020-02-16

**Authors:** Erminia Mariani, Lia Pulsatelli

**Affiliations:** 1 Laboratorio di Immunoreumatologia e rigenerazione tissutale, IRCCS Istituto Ortopedico Rizzoli, Via di Barbiano 1/10, 40136 Bologna, Italy; lia.pulsatelli@ior.it; 2 Dipartimento di Scienze Mediche e Chirurgiche, Alma Mater Studiorum-University of Bologna, Via Massarenti 9, 40138 Bologna, Italy

**Keywords:** platelet-rich plasma, platelet-rich fibrin, preparation, composition, musculoskeletal diseases

## Abstract

Platelet concentrates (PCs), mostly represented by platelet-rich plasma (PRP) and platelet-rich fibrin (PRF) are autologous biological blood-derived products that may combine plasma/platelet-derived bioactive components, together with fibrin-forming protein able to create a natural three-dimensional scaffold. These types of products are safely used in clinical applications due to the autologous-derived source and the minimally invasive application procedure. In this narrative review, we focus on three main topics concerning the use of platelet concentrate for treating musculoskeletal conditions: (a) the different procedures to prepare PCs, (b) the composition of PCs that is related to the type of methodological procedure adopted and (c) the clinical application in musculoskeletal medicine, efficacy and main limits of the different studies.

## 1. Introduction

In the last 10 years, autologous biological blood-derived products have been largely investigated as useful therapeutic tools for treating musculoskeletal conditions (such as osteoarthritis, muscle injuries, tendinopathies and intervertebral disc degeneration) [1,2,3]. Platelet concentrates (PCs), mostly represented by platelet-rich plasma (PRP) and platelet-rich fibrin (PRF), are included in this type of biology-oriented autologous therapeutic strategy that may combine plasma/platelet-derived bioactive components (cytokines, chemokines, growth-factors and enzymes) with fibrin-forming protein able to create a natural three-dimensional scaffold [4].

This approach allows us to deliver biomolecules released by a concentrated pool of activated platelets to the target tissue site of injury, thus effectively contributing to the modulation of inflammatory process, angiogenesis and immune response, as well as promoting the healing and repair of injured tissues [5,6]. Moreover, biological blood-derived products have been recognized to have antimicrobial effects, such as being able to inhibit and/or to inactivate different bacterial strains [6,7,8].

The potential clinical application of these biologic products in musculoskeletal medicine relies on their capability of modulating the joint environment and their beneficial role in reducing the local inflammation and promoting cartilage and synovium anabolism [5,9,10,11,12].

These types of therapeutic strategies provide advantages in clinical applications due to the autologous-derived source, safety profile, easiness to obtain and the minimally invasive application procedure. On the other hand, clinical efficacy is still controversial, and solid evidence and consensus supporting the therapeutic application are still to be achieved.

Indeed, there are already some issues to be addressed concerning the high variability of platelet concentrate products, which mainly depends on patients’ characteristics (age, sex, circadian rhythms and drug regimen) [13,14,15,16], as well as on the lack of standardized methods for platelet isolation/collection/activation and on heterogeneity among therapeutic protocols applied in clinical practice.

In this narrative review, we focus on three main challenging topics concerning the use of platelet concentrate for treating musculoskeletal conditions: (a) the different procedures to prepare platelet concentrate, (b) the composition of these products that is mainly related to the type of methodological procedure adopted and (c) the clinical application in musculoskeletal conditions and level of efficacy.

### Short History of Platelet Concentrates

The concept of PRP originally was developed in transfusion medicine. In this field, the PRP term was used in 1954 by Kingsley [17] to identify thrombocyte concentrate for treating patients with severe thrombopenia.

The history of the techniques to obtain blood-derived products for improving tissue healing started in 1970 with the studies of Matras [18] on fibrin glue use in a rat model.

Subsequently, an autologous product termed “platelet–fibrinogen–thrombin mixture” was developed, including, in fibrin glue, a significant concentration of platelets, in order to reinforce the fibrin polymerization [19].

In the following years, the role of platelets in supporting tissue healing was confirmed and clinically demonstrated by using a blood-derived product called “platelet-derived wound healing factors or formula-PDWHF” [20] for treating skin ulcers.

About ten years later, Whitman et al. [21] published a clinical study on the results obtained in oral and maxillofacial surgery by using a “platelet gel” obtained by a gradient density cell separator.

However, the term of PRP in regenerative medicine associated to the notion of platelet growth factors to promote tissue healing was truly introduced by Marx et al. in 1998 [22], in a study that reported the effect of platelet-rich product on bone healing in maxillofacial surgery.

After these publications, the term “PRP” was generically associated with all the multiple formulations of platelet concentrates. Afterward, an end-product characterized by a fibrin matrix denser and more stable than in other PRP formulations was produced and called platelet-rich fibrin matrix (PRFM) or pure platelet-rich fibrin (P-PRF).

In 2001, a different form of platelet concentrates was proposed and identified as leukocyte- and platelet-rich fibrin (L-PRF) [23]. These preparations are organized as a high-density fibrin and were considered as a “second generation” platelet concentrates. This family of platelet concentrates appears to be particularly suitable for oral clinical application.

## 2. Preparation Procedure

### 2.1. Platelet-Rich Plasma

PRP is obtained from autologous blood by using commercial kits or “in-house techniques”, aiming to provide a product characterized by a supra-physiological platelet concentration that can be used as liquid or activated gel form [14,24,25,26].

Despite the broad spectrum of protocols for PRP preparation, a common sequence of key steps [27,28] can be identified involving peripheral blood drawing from the patients by venipuncture, blood centrifugation to retrieve platelet-enriched fraction and platelet stimulation to release bioactive molecules.

In each of these phases, potential sources of variability may be identified, mainly ascribed to volume of blood samples drawn, type of anticoagulant, centrifugation protocols, material of collection tubes and type of platelet-activating agents [14,24].

The great variability in the different procedures results in a wide heterogeneity among PRP preparations in terms of platelet concentration, presence/absence of leukocytes and erythrocytes, and ultimately in terms of biological potential [14,24].

#### 2.1.1. Anticoagulants

There are multiple choices of anticoagulants (ethylene diamine tetra-acetic acid-EDTA, citrate dextrose-A, tri-sodium citrate and heparin) that are used for blood collection and that can differently affect PRP quality [29].

Lei and colleagues [30] investigated the effect of heparin, citrate, acid citrate dextrose (ACD) and citrate-theophylline-adenosine-dipyridamole (CTAD) on platelet-rich plasma quality, to determine the appropriate anticoagulants for PRP production.

ACD and CTAD appear to be more effective compared to heparin and citrate in maintaining the integrity of platelet structures and in preventing their spontaneous activation. ACD-PRP and CTAD-PRP released more TGF-beta1 and significantly increased the proliferation rate of human marrow stromal cells compared to heparin- and citrate-PRP, thus showing ACD and CTAD appropriate anticoagulants for PRP production [30].

An animal model study, aiming to investigate the influence of sodium citrate and ACD- solution A anticoagulants on cell count and growth factor concentration in pure platelet-rich gel supernatants, reported an increased number of platelets and leukocytes in sodium citrate PRP compared to homologous acid–citrate–dextrose solution A PRP fraction, but no difference concerning growth factor concentration [31].

Another “in vitro” study explored the effects of sodium citrate (SC), EDTA, or anticoagulant ACD- solution A, on PRP characteristics and on mesenchymal stromal cell (MSC) culture [29]. A higher platelet count was observed in blood collected with EDTA, even if an increase of mean platelet volume has been reported after the two centrifugation steps. Conversely, following the centrifugation procedure, platelet yield was higher in SC product. SC and ACD showed similar efficacy in inducing MSC proliferation [29].

These findings support the most frequent use of citrate-based anticoagulants for PRP preparations [29].

A very recent comparative study [32] evaluated the effects of EDTA, heparin sodium (HS) and SC on PRP quality and on bone marrow stem cells’ functionality.

Compared to HS and SC, EDTA has been shown to preserve platelet structure, minimize their spontaneous activation and sustain growth factor release for a more extended time.

Overall, these findings underline that also the choice of the best anticoagulant represents an open issue to address for optimizing PRP formulation.

To overcome this criticism, a study published in 2018 described a novel approach of PRP preparation without any additive, named temperature controlled PRP (t-PRP), by which the coagulation was previously inhibited in hypothermic environment. In this study, t-PRP was compared to PRP obtained by ACD-A blood.

Overall, t-PRP showed a more physiologic pH, higher platelet yield, slower release and degradation of growth factors. Furthermore, animal model experiments demonstrated that t-PRP was able to promote wound healing [33].

#### 2.1.2. Isolation Protocols

PRP can be obtained according to two basic protocols designed as plasma-based and buffy-coat-based procedures [14,34]. Plasma-based methods retrieve platelets, while minimizing leukocyte and erythrocyte fractions. For this purpose, a slower and shorter spin regimen is applied in plasma-based protocols. Platelets concentration is usually twofold to threefold increased above baseline whole blood levels (300,000 to 500,000 platelets/μL) [14,34].

Alternatively, the main goal of protocols for buffy-coat systems is to maximize platelet isolation during the centrifugation procedure, by high spin rates and long spin regimens. PRP obtained by this method is characterized by a high platelet recovery, increasing about threefold to eightfold compared to baseline levels (500,000 to 1,500,000 platelets/μL) and by the presence of variable concentrations of leukocytes and erythrocytes [14,34]. This type of PRP preparation is generally called leucocyte-rich PRP (L-PRP).

Specific protocols developed to obtain PRP by using either a commercial device/kit or manual/homemade procedures derive from multiple modifications of these two basic protocols (plasma-based and buffy-coat-based).

Most of commercially available systems produce PRP by buffy-coat-based method [35], and several comparative studies were reported, aiming to analyze different common commercial separation systems, essentially evaluating final PRP products in terms of platelets concentrations and growth factors release [24,35] (Table 1).

As expected, overall findings underlined that commercially buffy-coat-based systems (such as SmartPrep, GPS III and Magellan systems) yield higher concentrations of platelets and leukocytes compared to plasma-based systems (such as ACP and Cascade). Among buffy-coat systems, generally, GPS III preparations demonstrated the highest concentration of platelets and leukocytes [35].

Wide variations of centrifugal force and total centrifugation time among the different common commercial systems were described, respectively, ranging from about 350 to 2000 g, and from 5 to 20 min [35]. The majority of the systems use a dual-spin method; the first centrifugation usually has a lower speed compared to the second one [24,35].

Conflicting results were reported concerning optimal centrifugation rate to maximize platelet concentration, avoiding their activation or damage. Indeed, there is evidence underlining that increasing centrifugation force results in higher platelet concentration [37]. Conversely, other studies reported an inverse relationship between platelet yields and gravitational force [38,39]; furthermore, an elevated centrifugal speed could induce platelet activation [40].

Very recently, Croisè et al. [40] performed a literature review, aiming to check multiple studies focused on PRP protocol optimization. Fourteen included studies were commented upon, and each of them suggested different centrifugation procedures in terms of speed and duration time, number of centrifugations and, consequently, variable platelet concentration enrichments (from no enrichment to about 8.5 times more than peripheral blood). Overall these results underline that, to date, there is no consensus on the optimal centrifugation regimen to obtain a good-quality PRP, in terms of best platelet yields, avoiding structural and/or functional alterations and optimal relative concentration of blood components.

Recently, in order to obtain a standardized PRP formulation, Gato-Calvo et al. [41] developed a novel methodology, defining the optimal content of PRP, based on absolute platelet concentration. This approach allows us to obtain an end-product not influenced by the variability of the donor basal platelet counts, thus improving the reproducibility of PRP effects.

Another source of variability may derive from the material of blood-collection tubes. Some studies have demonstrated that PCs obtained by blood collected in glass or silica-coated tubes presented different buffy-coat morphology, fibrin architecture and platelet/leukocyte distribution in the PC matrix [42]. Furthermore, silica micro-particles may be released by tube walls during centrifugation procedures, entrapped in PC matrix, thus modifying platelet distribution in the end-product [43].

#### 2.1.3. Activation Process

Activation triggers two responses during PRP preparation: the release of the bioactive molecules stored in platelet alpha-granules, and the matrix formation by fibrinogen cleavage [44]. Clot formation entraps released growth factors (GFs), thus enabling bioactive molecules to be delivered and confined at the injured target site.

Activation process may be induced by endogenous and exogenous factors. Among exogenous factors, the most common activators are thrombin, calcium chloride and a mixture of calcium chloride plus thrombin [14,34,45]. Endogenous activation relies on the exposure of native collagen or other coagulation factor (such as adenosine diphosphate-ADP, thrombospondin and platelet-activator factor), spontaneously inducing clot formation at injured site [45].

In general, thrombin triggers a rapid platelet aggregation and stimulates a fast release of GFs [14,34,46]. Calcium chloride and collagen sustain a slower long-term release [34,46,47]. Furthermore, some findings reported that collagen activation results in a lower amount of released GFs compared to thrombin and calcium chloride [47].

A very recent study compared the effects of three different activation factors, thrombin, collagen I and ADP, on PRP quality and on bone marrow stem cells’ (BMSCs) functionality. Collagen I-PRP has been shown to induce the most rapid increasing of BMSC number compared to the rate observed with ADP- or thrombin-activated PRP. In addition, BMSC seeded in Collagen-I-activated PRP induced a significantly higher gene expression of osteogenic differentiation markers, osteocalcin and RUNX2, compared to thrombin and ADP. Thrombin induced a rapid and direct GF release, while collagen-I-activated PRP showed a sustained and slow GF release. The lowest total release was observed for ADP-activated PRP [32].

The different kinetic release is a crucial issue that might influence the availability of bioactive molecules, so affecting treatment outcome. Indeed, given GFs’ short half-life (from minutes to hours), if they are not promptly used upon platelet release, their degradation may occur before additional receptors, that are involved in the repair process, become available on cell surfaces [34].

Photo-activation has been suggested as an alternative method to trigger platelet activation: a very recent paper [48] described in vitro characterization of platelet photo-activation (polychromatic light source, in the range near-infrared region), in comparison with resting platelets and calcium chloride mediated PRP activation. That study showed that photo-activation of PRP induced a significantly more prolonged release and higher amount of platelet-derived growth factor (PDGF), basic fibroblast growth factor (FGF), and transforming growth factor (TGF)-beta than PRP activated with calcium chloride. Future clinical studies should be performed to verify the potential of using the photo-activation approach in PRP formulation.

### 2.2. Platelet-Rich Fibrin

This type of PC essentially includes two categories of different preparations organized as a high-density fibrin solid form: leukocyte-poor or pure platelet-rich fibrin (P-PRF) and leukocyte- and platelet-rich fibrin (L-PRF) [25,49]

Concerning P-PRF preparation, there is only one formulation, commercially known as Fibrinet (Platelet Rich Fibrin Matrix-PRFM, Cascade medical, Wayne, NJ, USA,) [25,49]. P-PRF is obtained by a double-centrifugation method analogous to other PRP protocol, but it differs since the clotting phase is a dynamic process occurring during the second centrifugation, after adding CaCl_2_ [25,49].

L-PRF is a leukocyte-rich product, and compared to PRP, L-PRF preparation is easier and lacks biochemical modifications (no exogenous activation or anticoagulant are required), and unlike PRP, PRF end-products are characteristically organized in tridimensional architecture [25,49].

L-PRF protocol was developed by Choukroun et al. [23] as an open-access technique, based on one-step centrifugation without anticoagulant and blood activators. L-PRF is considered to be a second- generation platelet concentrate [25,50]. Briefly, venous blood collected in glass tube without anticoagulants is centrifuged at low speed, and clot formation is immediately triggered. Three layers become evident after centrifugation: the red blood cells (RBCs) bottom layer, a PRF clot in the middle and the acellular plasma top layer [50].

This procedure allows to harvest almost all the platelets and more than 50% of the leukocytes from the peripheral blood [50]. L-PRF clot appears to be organized in a strong fibrin architecture and presents a specific tridimensional distribution of the platelets and leukocytes [50].

The original open-access experimental method has evolved into a regulated medical device system and is marketed with CE/FDA clearance (Intra-Lock, Boca-Raton, FL, USA). This system is the only certificated L-PRF system available on the market, and it uses the original protocol and devices [51]. This method shows a high efficiency in platelet and leukocyte collection and in leukocyte preservation [25].

Many variations of the original method were proposed, using different centrifuges and/or different protocols. These modifications result in modified-PRF product compared to the original L-PRF.

P-PRF procedure is more expensive and complex compared to L-PRF protocol. Furthermore, this latter procedure allows to simultaneously obtain a large number of end-products [25].

To the best of our knowledge only one paper [52] compared PRFM and PRF products, in terms of growth factor release. In this study, PRFM and PRF were obtained by “home-made” protocols and appeared to have a different kinetic release. PRFM presented an early robust boost of growth factors, while PRF release was more gradual and constant up to 23 days. On the contrary, Lucarelli et al. [53] has shown that Fibrinet PRFM releases elevated levels of growth factors (such as PDGF, TGFβ and VEGF) in the first 24 h, whereas other growth factors, such as bone morphogenic protein (BMP)-2 and -7 were undetectable.

Conversely, L-PRF products sustained a large growth factor release for up to seven days [50]. Interestingly, BMP-2 was detected in L-PRF releasate strengthening the regenerative potential of this PC [51]. It is hypothesized that the presence of leukocytes may have a relevant impact on the amount and the pattern of the released growth factors, and a potential synergistic effect between leukocytes and platelets has been suggested [25,50,51].

Centrifuge characteristics and centrifugation protocols have been shown to impact fibrin architecture, cellular distribution and growth factor release. Therefore, various PRF preparations could be associated to different biological profile and clinical potential [51]. Up to now, the different PRF preparations are not clearly characterized, and further investigations on the effects of protocol modifications need to be provided.

## 3. Classification Systems

The heterogeneity of PC preparation methods can impact on the functional characteristics and on the potential therapeutic efficacy of the final products, giving each PC formulation unique properties. The majority of the studies do not provide a full characterization of the various PC composition, so a reliable comparison among studies still remains a challenging issue [54].

Several classification systems (Table 2) have been developed over the years in attempt to help comparison among studies and to foster standardization of PC preparation process. However currently, no consensus on classification systems has yet been achieved [54].

## 4. Composition

### 4.1. Platelets

The human blood platelet normal concentration ranges from 150,000 to 400,000/µL [61]. There is no consensus on the optimal concentration of platelets in PCs.

Platelet concentration was compared for its healing effect, and different optimal levels were identified for different applications [14,34].

PRP platelet concentration greatly differs in PRP obtained by the various commercial systems.

Plasma-based PRP systems usually contain a platelet concentration between baseline and 3x baseline (less or equal to 750 × 10^3^ platelets/µL), and they are defined as low-yielding devices (such as ACP, Cascade, Endoret and RegenPrep) [35]. On the other hand, buffy-coat-based systems yield platelet concentration above 3x, ranging from 4x to 6x (greater than 750 × 10^3^platelets/µL to 1800 × 10^3^ platelets/µL). These systems are classified as high-yielded devices that produce PRP (GPS III, SmartPrep and Magellan) [35].

In vitro, in vivo and clinical studies have demonstrated successful results for PRP formulations with both a moderate (2× and 3×) and high platelet concentrations (from 4× to 6×) [14]. In particular, an in vitro study evidenced that the best angiogenic effect of PRP was obtained with 1500 × 10^3^ platelets/µL, thus underlining the role of platelet concentrations on the clinical application when the increased angiogenesis contributes to the healing process [14,62].

Platelet concentration greater than 6x (>1800 × 10^3^ platelets/µL) may be detrimental or have side effects [63]. In fact, an excessive platelet amount may lead to cellular apoptosis, downregulation and desensitization of growth factor receptors, resulting in a paradoxical inhibitory effect [34].

Another source of variation is the platelet-counting mode. Indeed, it has been reported that, to achieve accurate platelet count, proper sample preparation is required and manual mode in the hematology analyzer is recommended, because automatic mode, allowing the sample to settle, may underestimate the absolute platelet count [34,64].

### 4.2. Leukocytes

As previously stated, leukocyte content in PCs depends on PRP preparation procedures.

Plasma-based process reduced leukocyte count up to 22 times the baseline, almost eliminating this cellular fraction. Buffy-coat-based procedures actively concentrate leucocytes from threefold to fivefold the baseline [65]. Furthermore, different buffy-coat methods produce a PRP formulation with different proportions of neutrophils, lymphocytes and monocytes [65]. Indeed, it has recently been reported that different centrifugation regimens, in terms of spin numbers and speed, modified lymphocyte/granulocyte ratio in the final products [66].

The inclusion of leukocytes in PC preparations remains a widely debated concern, as both beneficial and detrimental effects have been suggested.

Deleterious effects are mainly ascribed to leukocyte capacity to release inflammatory cytokines and metallo-proteinases, which can promote pro-inflammatory and catabolic effects on targeted tissue [67,68,69,70]. Furthermore, the massive release of reactive oxygen species by neutrophils causes tissue damage, by inhibiting healing process [71,72].

On the other hand, potential beneficial effects rely on leukocyte’s role in tissue healing, in regulating inflammatory process [73,74,75] and in antibacterial activity [76,77] that may switch the inflammatory process toward a regenerative phase.

These potential effects are suggested and corroborated by the following main evidence:

- The presence of leukocytes contributes to potentiate total amount of released GFs [35]. Indeed, several studies have reported a positive correlation between leukocyte count and GF concentration [35,78,79,80].
- Leukocytes have anti-nociceptive action by releasing anti-inflammatory cytokines (IL-4, IL-10 and IL-13) and opioid peptides (beta-endorphin, Met-enkephalin and dynorphin-A) [25,81].
- Circulating monocytes differentiate into macrophage once they migrate into connective tissue and may switch from M1 (pro-inflammatory) to M2 (anti-inflammatory) phenotype [82,83] in response to micro-environmental signals and stimuli (such as neutrophil-derived micro-vesicles [4,84]).
- M2 macrophages have several functions in tissue remodeling, promoting angiogenesis, cell proliferation and extracellular matrix deposition [83,85], and they may contribute to resolution of inflammation [4].
- Proteinases secreted by leukocytes are able to modulate the activity of secreted growth factors, converting inactive form to active one and contributing to matrix remodeling in tissue healing [71,75].
- Neutrophils are essential for killing bacteria and other microorganisms [86]. Since platelets also contribute to the antibacterial response [6,7,8], leukocytes may synergize with platelets and potentiate PRP antimicrobial effects.

Furthermore, growing evidence on the relevance of leucocyte–platelet interaction and of their relative proportions in PRP preparation has been reported [4,44,50,66,87]. Indeed, leucocyte–platelet interaction may promote biosynthesis of other factors that facilitate the resolution of inflammation, such as lipoxins that are potent anti-inflammatory proteins able to limit neutrophil activation, so promoting the resolution phase of the healing process [44,88,89].

In addition, the interrelationship between platelets, blood cellular components and fibrin may have a key role in proper platelet function and growth factor release [4,50,87], and the relative platelet/leukocyte and lymphocyte/granulocyte ratios might drive the balance between catabolic and anabolic factors [66].

Therefore, future research efforts should not focalize on the concentrations of single PC component but on the optimal relative combination of platelets, leukocytes, growth factors and fibrin within the final preparation for the different clinical application fields.

### 4.3. Red Blood Cells

Red Blood Cells (RBCs) can be damaged as a result of high shear force during blood collection or during inadequate centrifugation process, so causing hemolysis with the release of hemoglobin and its degradation products, hemin and iron. The presence of these hemolytic-related products lead to several deleterious effects, such as radical oxygen reactions, endothelial disfunction, vascular endothelium damage, pro-inflammation response and tissue injury [90].

RBC damage also causes the release of migration inhibitory factor (MIF), which has been recognized as a very strong inflammatory cytokine [90]. MIF concentration in whole blood is 1000-fold increased than in plasma. Since leukocytes and platelets have been shown to minimally contribute to MIF concentration, RBCs represent the major reservoir of this factor [91], which is also functionally active [91].

MIF plays a pathophysiological role in promoting and maintaining OA pain [92]. Furthermore, MIF levels in plasma and synovial fluid have been found to be positively correlated to disease severity in knee OA [93]. Blood-induced joint damage has been highlighted by various in vitro studies. In fact, blood exposure results in increased synoviocyte cell death and pro-inflammatory mediator production [94], induction of chondrocyte apoptosis and cartilage degradation [95,96,97].

On the other hand, effects of free heme may be inhibited by its degradation or by specific binding proteins. The heme–heme oxygenase (HO) system is formed after HO-mediated heme degradation. Growing evidence support the protective HO system activity and its effector molecules against oxidative and inflammatory responses and cell damage and suggest that the heme-HO system may represents a novel and important target in the control of wound healing [98,99,100].

Even if RBC content is reduced or absent in PC preparations, the detrimental effect of RBCs should be addressed for optimization of PC performance.

### 4.4. Growth Factors

GFs and protein are stored in the platelet alpha-granules and are released by activation of the platelets. Over 300 proteins were identified in the platelet releasate [101].

Multiple pieces of evidence have suggested that platelet-derived growth factor (PDGF), transforming growth factor beta (TGF-beta), vascular endothelial growth factor (VEGF), insulin-like growth factor (IGF) and epidermal growth factor (EGF) are the most crucial factors implicated in tissue repair [102]. PDGF, TGF-beta and VEGF appear to be the most investigated, and the concentration of these GFs is often considered as a marker of PC preparation quality [24,35,102].

In PRP preparations, approximately 70% of platelet growth factors are secreted within the first 10 min following activation, and almost 95% within the first hour [103,104]. Platelets may continue to produce small amounts of growth factors during the residual life span (8–10 days) [103,104]. Conversely, PRF presents a more intense, slow and constant long-term release, up to 5–7 days [50,105].

Together with platelets, leukocytes also contribute to the release of some growth factors, as highlighted by several studies that reported a positive correlation between the amounts of released GFs and the number of leukocytes [35,78,79].

Multiple comparative studies have investigated GF released by PRP obtained by various commercial separation systems. A large heterogeneity in the GF concentrations and kinetic release have been shown when comparing multiple PRP preparations obtained by different commercial separation systems [106].

A recently published review underlined that growth-factor concentrations reported by the different studies appeared to be hardly comparable, due to wide variations of these results, not only among the different systems but also when comparing the same separation systems among the different studies [35]. This variability may be essentially ascribed to two criticisms: the different commercial kits used for growth-factor dosage [35], and the incomplete removal of platelets and erythrocytes that may impact the results [107]. Due to these limitations, a comparison between studies appears to be barely reliable, not allowing consistent evidence-based results concerning growth factor content profile of different PRP preparations.

Furthermore, the great inter-individual variability of GF concentration needs to be taken into consideration [107,108]. A study performed on a large number of OA patients (n = 105) showed a wide individual variation of PRP growth factors, with a coefficient of variation ranging from 5.30 to 78.45. In particular, basic FGF and TGF-beta1 showed, respectively, the highest and the lowest variation [109].

Concerning PRF, different GF releases by different formulations have been shown. Comparing original L-PRF to modified-PRF formulations, conflicting results were reported. Kobayashi et al. [105] demonstrated that significantly higher GF levels were released by advanced-PRF (A-PRF) compared to original L-PRF. On the other hand, Dohan Ehrenfest et al. [51] reported a much stronger release of GFs from original L-PRF than from A-PRF membrane.

Nowadays, the literature data highlights that biological profiles in terms of content, amount and release kinetics associated to different PCs need to be further investigated, in order to better understand GF potentiality of the various PCs in clinical applications.

## 5. Clinical Efficacy

### 5.1. Osteoarthritis

Osteoarthritis (OA) is a debilitating osteo-articular disease, triggered by a trauma to the joint, and it is associated with a progressive erosion of articular cartilage, subchondral bone sclerosis, excessive stiffness and pain.

Numerous clinical trials and case series, carried out using PRP administration in patients with OA, supported PRP for the symptomatic effect, reduction of pain, improvement in the degenerative injuries and safety of administration, but they have not reached an univocal consensus.

#### 5.1.1. Knee Osteoarthritis

Knee OA is a chronic disease of joints that is characterized by pain and progressive disabilities, usually developing as the sufferer ages [110]. The most common treatments, both non-pharmacological and pharmacological, show positive outcomes, but their effectiveness is not long-lasting. Thus, surgical knee replacement is often the last chance for the relief of symptoms [111,112]

One of the first PRP studies establishing the safety of intra-articular use of this autologous preparation dates back to 2008 [113] (Table 3).

Afterward, different studies demonstrated the positive effects of PRGF/PRP injection, either when used alone or when compared to hyaluronic acid (HA) one, in the knee OA patients [114,115,117,119,120,121,122,123,124,125,126,127,128,129,130,131,132,133,134,135,136,137,138,139,140]. These PCs were reported not only to have an effect on clinical symptoms (by decreasing pain and improving function), but also on synovial fluid and protein amounts, as well as on cartilaginous degeneration.

However, a recent study reporting results of a follow-up up to six years does not confirm superiority of PRP [142].

The superiority of PRP was also established by comparison with normal saline (physiological control), as indicated by early improving WOMAC (the Western Ontario and McMaster Universities Osteoarthritis) scores, and maintained up to six months [153,158,163], but slightly decreased afterward, in agreement with the anti-inflammatory action supposed for PRP [172].

Similarly, in a trial including 366 young patients (18–30 years old), positive outcomes were reported after intra-lesional PRP administration [162]. In general, better results were obtained in young patients, with low body mass index [117,122].

PRP was reported as better in terms of clinical improvement compared to oral NSAID administration [161], as synergistic and protective, when added to methylprednisolone [150] and comparable to HA and corticosteroids after three months, superior to both the other treatments in the long-term [149].

Both PRP and HA have a biological origin and may be critical for tissue healing at the beginning of OA development. In in vitro studies, the combination of PRP with HA may display synergistic effects on fibroblast migration [173,174], thus suggesting a better effect of PRP–HA combination than PRP alone [175].

In agreement, a recent randomized clinical trial in mild/moderate knee OA reported better outcomes of the patients treated with PRP–HA combination when compared to PRP (up to three months) or to HA (up to 12 months) groups [144].

Furthermore, the synergy between combined PRP and HA treatment was further investigated and compared with each of them alone and with a placebo, via intra-articular injections in a total of 360 patients with knee osteoarthritis [145], demonstrating significantly reduced pain and decreased immune response, as well as PRP treatment compared with low and high molecular weight HA [143].

Even if clinical studies on PRP–HA combined therapy are limited and there are several peculiar aspects of HA alone (such as molecular weight), of the PRP–HA mix (such as ideal combination and dosage schedule), the preliminary data are worth of being deepened.

The PRP administration schedule in OA knee, widely reported with different numbers of injections, different time intervals and duration, represents a further aspect to be defined.

Patel [153], first compared the effect of one with two PRP injections and showed similarly improved WOMAC scores. A following double-blind placebo-controlled randomized trial demonstrated that the patient group that had undergone three PRP injections presented a better score than groups treated with a single dose of PRP or HA [154].

A clinical efficacy of PRP was also described when PRP was alternatively used at annual intervals or at the request of the patient when the effect ended [123]. Moreover, the administration in two phases foreseeing six doses at weekly intervals, and then a three month suspension and a maintenance dose (three injections at three-month intervals), presented interesting functional improvements [152].

A single administration of very pure PRP offered a significant clinical benefit as one injection of HA [140], and a similar improvement was obtained by a single administration of about 9 mL of PRP [155].

An enlarged delivery approach was also described, firstly for the treatment of severe OA [169] and more recently for the treatment of mild to moderate forms [170,171]. In these studies, the intra-articular injection of PRP was associated with concomitant intraosseous PRP injections into the subchondral bone, obtaining significant results.

A significant improvement of pain and functional scores, as well as decreases of the inflammatory response, were also obtained by the concomitant injection of PRP both intra-articular and in peri-meniscal soft tissue structures, thus widening the PRP effect on pes anserine tendons, bursa, medial collateral ligament and medial meniscus [176].

A systematic review on PRGF [177] reported the efficacy of PRGF in pain improvement, but also pointed out the limits of the included studies that prevented to perform a meta-analysis. The heterogeneity of the primary outcomes, PRGF and HA administration schedules, HA molecular weight, the small number of studies fulfilling the eligibility criteria and the lack of placebo treated group were the main drawbacks.

PRP was described as effective, alternative and superior to HA treatment for long-term improvement of joint function and pain in patient with knee osteoarthritis, mainly in early-moderate disease compared to advanced disease. The limits reported in a narrative review [178], in a recent meta-analyses [179,180] and in a systematic review [181] evidenced the variability of OA severity (K-L I-IV), as well as age, sex and BMI in patients treated in the different studies. In addition, main criticisms concerned the number of injections, optimal dosage of PRP, administration schedule, heterogeneous PRP preparations and formulation discrepancies, absence of published studies supporting specific protocols of injection and lack of indications on the appropriate regimen for different OA severity degrees. The limited size of pooled patients that can under-power the statistical analysis to reach a significant threshold of difference in outcome measures, and the lack of a placebo group shades the evidence of PRP effects.

#### 5.1.2. Hip Osteoarthritis

Although various trials have faced up to the use of on PRP use for knee OA, few studies have focused on the treatment of hip OA with PRP. These studies are summarized in Table 4.

A recent study [188] described the intraosseous infiltration of PRP for the treatment of hip osteoarthritis, in agreement with knee reported ones. Future studies are required to confirm the potential advantage of this new application of PRP.

Meta-analysis results of a randomized clinical trial that compared the effectiveness of PRP versus hyaluronic acid (HA) in hip OA underlined that PRP treatment was related to a significant reduction of VAS at two months. Both PRP and hyaluronic acid appeared to be comparable in terms of functional recovery [189].

The systematic review on the use of ultrasound-guided PRP injections in the treatment of hip osteoarthritis concluded that this route of administration appears to be well tolerated. Furthermore, though the level of evidence is relatively low, PRP treatment may lead to efficacious long-term and clinically significant reduction of pain and functional improvement [190].

Overall, intra-articular injection of PRP in hip OA patients has been demonstrated to be safe and have some efficacy in pain reduction and in functional improvement. When compared with HA, PRP showed to induce a better early pain relief; however, over 12 months, PRP and HA had comparable effects.

Future large-size trials that include a placebo group are needed. These studies should increase the level of evidence for the actual potential efficacy of PRP as an alternative conservative treatment to delay surgery in hip OA patients.

#### 5.1.3. Ankle Osteoarthritis

Osteoarthritis of the ankle is less common than the previously described localization of OA. Data concerning the use of PRP in ankle OA are obtained by case series. Four injections of PRP at weekly intervals induced improvement of function, pain and patient satisfaction [191], and similar improvements in pain and function up to 24 weeks after treatment were obtained after the administration of three injections every two weeks [192].

The limited data show some benefit in short–medium time, demonstrate the safety of the therapy and can be considered to be an alternative to postpone the need for surgery, but the comparisons with other injectable controls are lacking; therefore, no definitive conclusion can be made about the benefit of PRP in ankle OA.

### 5.2. Tendinopathies

Tendon tissue is poorly vascularized, and this characteristic is responsible for the limited healing capacity and the lesion irreversibility resulting in tendinopathies, which frequently occur in athletes [193].

#### 5.2.1. Achilles Tendinopathy

Achilles tendinopathy is a painful condition. Physical stress leads to tendon micro-trauma, and the inflammatory and degenerative responses that follow are responsible for local pain, swelling and stiffness [194]. Its treatment is difficult, and sufferers easily relapse due to the poor curative effects of the conservative treatment approach. The reason for PRP application lies in the tendency of the tendinopathy to became chronic after the use of nonsurgical approaches.

The outcomes after PRP administration are variable, and the main results are reported in Table 5.

Case series for chronic Achilles tendinopathy [195,196,197,198,199,200,211], retrospective studies [201,204] and prospective studies [208,209,210,215] have described promising efficacy of PRP treatment with lasting improvements [205].

Other studies did not show a superiority of PRP injection over saline solution [206,207,213] and no differences between patients treated with leukocyte-rich or -poor PRP [208]

Evidence for the efficacy of PRP in Achilles tendinopathy is not in agreement, and despite the important clinical significance, a strong basis for the use of PRP for Achilles tendinopathy was not demonstrated by meta-analyses and a systematic review [216,217,218,219].

#### 5.2.2. Lateral Epicondyle Tendinopathy

Lateral epicondyle tendinopathy, also known as “tennis elbow” is a common cause of pain and disability. Symptoms have been attributed to micro-trauma to extensor carpi radialis brevis tendon and the resulting angiofibroblastic tendinosis [220].

Different therapeutic approaches have been used, and steroid injections are considered to be the gold standard. Recently, PRP also became popular in treating this disease, with effects opposite to those of steroids, by stimulating the healing process and down-modulating inflammatory response.

The majority of the studies compared PRP efficacy with steroid one; however, other treatment comparisons have been reported (Table 6).

Initial results have been promising [221,222]. The first randomized controlled trials displayed PRP treatment improvements in function and pain, exceeding the effect of steroid injections up to one [232] and two [233] years

Following trials, comparing PRP treatment with saline [228,229,245], steroid [232,233,234,235,236,237,238,239,240,245] autologous whole blood [225,226,227] and bupivacaine [241] showed variable effectiveness in reducing pain and improving function.

Studies showing similar therapeutic effects between PRP and whole blood [225,226,227] suggest that circulating platelet concentrations are enough for obtaining recovery. However, the limited patient number and the absence of placebo arm make questionable these results.

As far as we know, the results of a multicenter randomized controlled IMPROVE trial are not yet available. The four-arms of lateral epicondylitis treatment will compare PRP, whole blood injection and tendon fenestration, each associated with physical therapy and sham superficial subcutaneous soft tissue injection, plus physical therapy. Expected results should significantly impact clinical practice [249].

Despite the heterogeneity of data, a seven-year retrospective study [250] and several meta-analyses, differing for inclusion criteria are available for evaluation the effectiveness of PRP in the treatment of lateral epicondylitis [251,252,253,254,255].

These reviews demonstrated short-term benefits for corticosteroids, but a long-term effectiveness for PRP in regard to improving functional capacity and alleviating pain. The critical factors identified mostly mirror those evidenced in other anatomical sites. Volume and number of administrations, various treatment combination, lack of standardization for PRP preparation and for exercise protocol, different measures for outcome evaluation and different follow-up times need deeper assessments.

#### 5.2.3. Plantar Fasciopathy

Plantar fasciopathy (PF), also known as “plantar fasciitis”, affects the proximal insertion of the plantar fascia in the os calcis, causing pain. Tissue thickening and degenerative structural changes are more common than inflammatory findings, so the “plantar fasciopathy” definition better identifies this disorder [256].

The fascia plays a role of primary importance in the transmission of body weight to the foot while walking and running. Plantar fasciitis is very common in athletes, but can also occur in overweight or obese subjects.

Corticosteroids, autologous blood injection and extracorporeal shock wave therapy (ESWT) represent treatment options that have been used with varying results.

At present, a uniform therapy for the management of Plantar fasciopathy is missing; therefore, many studies have considered PRP to be an intriguing alternative option to favor healing in the plantar fascia without significant risk [257] (Table 7).

Early cohort studies have described the positive effect of PRP injection on relieving pain [260] and improving function [259], as well as on tissue structure [258] for chronic plantar fasciopathy.

The most recent randomized controlled trials comparing PRP, corticosteroids and normal saline administration describe a similar or a superior effect of PRP compared to corticosteroid injection and normal saline in reducing pain and increasing functional scores for chronic plantar fasciopathy [278,279].

Numerous other studies obtained variable results by the comparison of PRP and corticosteroid treatments: PRP was described as being either able to favor early pain relief and functional improvement [267,275] with prolonged effects [266,269,271,274,278] or to be likewise effective up to six months [265,268,270,272,273,276].

Trials comparing PRP with other treatment options for plantar fasciopathy showed a better initial PRP response but similar effects at six months; when PRP was compared with prolotherapy [281], no significant differences compared to extracorporeal shockwave [280] or plasma injection [263], superior and long-lasting effects compared to KT [282].

The latest systematic reviews and meta-analyses comparing PRP to other therapeutic approaches supported the use of PRP for the lack of complications or side effects [284], but, above all, for its superiority to corticoids, especially in long-term pain relief [285,286]; however, small sample number, study heterogeneities, adverse events and the lack of recording PF recurrence following treatment may decrease reliability of outcome measures.

#### 5.2.4. Patellar Tendinopathy

Inferior pole patellar tendinopathy, generally known as jumper’s knee, is mostly common among athletes who engage in sports involving frequent jumping, such as volleyball and basketball, but it is also observed in people who do not carry out sporting activities [287]. The main evidence on PRP treatment in patellar tendinopathy is reported in Table 8.

PRP has been administered in several studies as a biological therapy for patellar tendinopathy, improving pain and MRI tendon structure, and significantly increasing functional outcomes, with long-lasting stable results up to two years, thus improving quality of life [196,199,288,289,290,303],

Multiple injections were found to be better than a single one for patellar tendinopathy, either in case series [293,294] or in a randomized prospective study [295], but the effect of two repeated injections or one single injection was also reported to be similar [296].

PRP treatment displayed better results than ESWT [299] and physiotherapy [297]. Dry-needling used for PRP administration made recovery faster than dry-needling alone; however, beneficial effects on pain and function only lasted three months, without improvement in QoL [301]. Furthermore, no clinical differences were observed when PRP was administered following other inefficacious treatments [298], or among leukocyte-rich or -poor PRP and saline [302].

Not long ago, no randomized controlled quality studies supported the use of PRP over conservative therapies, except in therapy-resistant cases [293,304]. However, recently, a systematic review [305] and meta-analyses of randomized trials have recommended the use of PRP for the management of patellar tendinopathy, due to its superiority to other nonsurgical therapies [306], in long-term pain relief and improvement in knee function [307]. Even if eccentric exercises seem to be the strategic choice in the short-term, in complexes cases, multiple PRP injections can be considered to be an option [308]. Variability on follow-up length, or its absence, and number of interventions are the main limitations of these studies.

### 5.3. Muscle Injuries

The use of PRP for the treatment of muscle injuries raised significant interest in the last years.

Similar to tendon healing, the initial muscle healing begins with an inflammatory response, followed by proliferation and differentiation of cells and tissue remodeling.

Acute hamstring injury is one of the most common muscle injuries affecting athletic patients, causing a decline in competition performance [309,310].

Some studies described positive results after injection of PRP in patients with injured skeletal muscles, and no negative side effects were reported [311,312] (Table 9).

Contrasting results were obtained when PRP was compared to saline [313,314,315].

In general, an earlier comeback to sports activity, together with lower scores of pain severity and no significant increase of the re-injury risk, has been observed in patients/athletes who have undergone PRP administration, combined with a rehabilitation program, compared to patients treated with a rehabilitation program alone [320,321,322].

In particular, as a randomized clinical trial, this study showed positive outcomes in the PRP group as concerning convalescence time and returning to play [321].

Despite some favorable results, these studies do not have enough statistical power to support evidence-based adoption of PRP administration for skeletal muscle injury in clinical practice, as recently widely debated [325,326]. In general, current clinical evidence are conflicting, and univocal findings on the efficacy of PRP injections in the treatment of muscle injuries have not been achieved. Therefore, further human studies are strongly required to assess and validate the effectiveness of PRP for skeletal muscle regenerative purposes.

Platelet growth factors, specifically myostatin and TGF-β1, have been shown to have harmful effects to muscle regeneration. Indeed, TGF-beta1 is involved in the regulation of the level of fibrosis during muscle-injury repair, which is an important link in the complete restoration of muscle function [327]. An vitro study [328] demonstrated that platelet-poor plasma (PPP) or PRP with a second spin to remove the platelets induced differentiation of myoblasts into muscle cells.

However, since experimental evidence has not received a large consensus [329,330], further studies are needed to define the exact PPP-growth-factor content, its effect on myogenic precursors and its role on skeletal muscle regeneration. In addition, human clinical trials will be required to further explore the potential beneficial effects of muscle injuries treated with PPP.

These overall findings underline that none of the therapeutic options so far adopted have led to reliable results [325,326]. Even if skeletal muscle tissue exhibits an intrinsic remarkable regenerative potentiality in response to injury, in the case of extended damage, a dysregulated activity of different muscle interstitial cells occurs, resulting in aberration of tissue repair and maladaptive fibrotic scar or adipose tissue infiltration [331]. In this context, the morpho-functional recovery of injured skeletal muscle still remains a scientific challenge, and the identification of strategies that efficaciously improve the endogenous skeletal muscle regenerative mechanisms represents an unmet need.

## 6. Conclusions and Future Perspectives

PC use has gained popularity for the treatment of musculoskeletal diseases, even if conflicting results have been reported concerning clinical efficacy. Inconsistencies of clinical results rely on the huge heterogeneity of PC preparations, mainly ascribed to individual characteristics, different preparation protocols and variability in composition, as well as on different methodological limits of the protocols adopted in the clinical studies that have been previously underlined.

In addition to the different critical aspects already considered, the indistinct employment of words to refer to fresh, frozen/thawed or activated preparations increases confusion. Therefore, also a simple aspect such as a classification nomenclature comprehensive of all PCs, with the same characteristics allowing an overall clinical outcome comparison, could contribute to define the clinical use and improve our knowledge of PRP.

Besides being a paramount component of PRP, platelets have been proposed as carriers of pharmacological or biological molecules [332]; therefore, “future” PRP could be implemented with suitable molecules favoring specific biological functions.

The possibility of encapsulating PRP with a combination of HA, gelatin and biodegradable scaffolds displayed interesting results in in vitro studies of bone regeneration [333], and a new delivery system linking fibrinogen with high molecular weight HA (RegenoGel™) (merging the respective regenerative/wound healing properties and viscoelastic characteristics) showed positive outcomes in mild/severe osteoarthritis. In addition, this system can be used as a carrier for microRNA or inhibitory molecules (ADAMTs), allowing the preparation of specifically targeted custom-made devices [334,335].

Encouraging in vitro and in animal model studies has demonstrated that PRP combined with different biomaterials prolonged and improved growth factor release [336]; however, the possibility to translate these engineered biomaterials in the clinical practice to develop novel therapeutic strategies remains a future perspective.

## Figures and Tables

**Table 1:** Centrifugation protocol and composition of platelet-rich plasma (PRP) produced by common commercial PRP systems.

Device	Centrifugation Force (g)	Centrifugation Time (min)	Platelet Concentration ×10^3^/µL	Leukocyte Concentration ×10^3^/µL	PDGF-AB pg/mL	TGF-β1 pg/mL	VEGF pg/mL
ACP	350	5	500	<1	3133–22,180	456–73,867	59–246,78
GPSIII	1100	15	273.6–1560	15–52	5900–65,000	2647–153,863	1304–1991
Cascade	1100/1450	6/15	600–2900	<1	6100–13,300	20–180	0–600
SmartPrep	1250/1050	14/7–10	800–2600	<1–20	123,100–293,500	22,400–132,000	/
Magellan	610/1240	4/6	600–1500	8–35	23,700–45,100	100–300	400–2000
JP2000	1000/800	6/8	850	26.1	93,500	1563	42,000
GLO	1800/1800	3/6	891	10	67,300	1329	39,000
KIOCERA	600/2000	7/5	1312	14	76,200	1508.2	44,000
Selphyl	525	15	88	0.3	12,200	384	28,200
MyCells	2054	7	800	4.9	72,200	1328	39,200
Dr Shin’s System	1720	8	650	14.9	37,000	938	31,000

Data (cumulative range or average) were obtained from the following references: [24,35,36].

**Table 2:** Summary of classification systems for platelet concentrates (PCs).

Study	Classification	Parameters
Dohan Ehrenfest et al. (2009) [25] (2012, 2014) [49,55]	Pure PRP, Leukocyte-rich PRP; Pure PRF, Leukocyte-rich PRF	Leukocyte content Presence/absence of fibrin
DeLong et al. (2012) [34]	PAW (Platelet Activation, White blood cells)	Platelet absolute number (from baseline to above 1250 × 10^3^/µL Activation method White Blood Cells and neutrophil content (above/below baseline)
Mishra et al. (2012) [56]	Sports medicine classification of platelet rich plasma.	Platelet concentration (< or ≥5 times baseline) White Blood Cell presence/absence Activation or no activation prior to application
Mautner et al. (2015) [57]	PLRA (Platelet Leukocyte Red blood cells and activation)	Platelet count (absolute number/µL) Leukocyte content (as positive/negative) Percentage of neutrophils Red Blood Cells contents (as positive/negative) Activation (yes or no for exogenous activation)
Magalon et al. (2012) [58]	DEPA (Dose of platelet Efficiency, Purity and activation)	Dose (platelet number × PRP volume) Efficiency (proportion of platelet recovery) Purity (proportion of platelet compared with Red Blood Cells and leukocytes) Exogenous activation (yes/no)
Lana et al. (2017) [59]	MARSPILL (Method, Activation, Red blood cells, Spin, Platelets, Image guidance, Leukocytes and Light activation)	Method (automated manner or manually) Number of spins Platelet concentration (Fold basal) Leukocyte content (< or ≥15 times baseline) Red Blood Cell content (< or >baseline) Photo-activation (yes/no) Image guidance (yes/no)
Harrison P (2018) [60]	ISTH (International Society on Thrombosis and Hemostasis) classification	Activation Platelet count (<900 × 10^3^ µL; 900–1700 × 10^3^ µL; >1700 × 10^3^ µL) Preparation method Leukocyte contents (as positive/negative) Red Blood Cells contents (as positive/negative)

**Table 3:** Evidence of PRP treatment in knee OA (reported by year of study and grouped by treatment).

Treatment/ Control	Reference	Patient Number	Main Results
PRGF/HA	Sanchez et al. [113]	60	Significantly higher rate of response to PRGF than HA treatment as concerning knee pain, stiffness and physical function scores, up to 24 and 48 weeks
	Sanchez et al. [114]	176	
	Vaquerizo et al. [115]	96	
	Raeissadat et al. [116]	69	No difference between PRGF and HA treatments in alleviating pain and improving function
PRGF	Wang-Saegusa et al. [117]	261	Improvement in function and QoL were described
PRGF (1 cycle)/PRGF (2 cycles)	Vaquerizo et al. [118]	48	PRGF 2 cycles showed improved stiffness and QoL, but not pain decrease
PRP	Kon et al. [119]	100	Significant improvement of knee pain, function and QoL during therapy; improvement has been described to last for a short (2 months) or a medium/long period (6–12 months) follow-up and subsequently worsen; however, the improvement remained higher than the basal condition; further improvement at 18 months can be obtained by yearly repetition of PRP injection; better results were obtained in younger patients, lower degree of cartilage degeneration and short disease duration; worse results were observed in over-80-years-old patients; PRP injection was associated with inflammation decrease and anti-ageing physiological function increase; improved symptoms and pain were not dependent on the cartilage damage degree, as determined by MRI
	Filardo et al. [120]	90	
	Gobbi et al. [121]	50	
	Halpern et al. [122]	22	
	Gobbi et al. [123]	93	
	Hassan et al. [124]	20	
	Bottegoni et al. [125]	60	
	Chen et al. [126]	24	
	Huang et al. [127]	127	
	Fawzy et al. [128]	60	
	Taniguchi et al. [129]	10	
	Burchard et al. [130]	59	
	Socuoğlu et al. [131]	42	
PRP/HA	Cerza et al. [132]	120	PRP compared with HA showed better clinical outcomes and QoL; clinical improvement was evident at 3–6 months and up to 12 months of follow-up; PRP treatment was effective in initial stages/low grade of knee OA but not in patients with grade III arthrosis; in middle-aged subjects with moderate OA, PRP and HA induced similar improvements
	Filardo et al. [133]	109	
	Spakova et al. [134]	120	
	Say et al. [135]	90	
	Guler et al. [136]	132	
	Raeissadat et al. [137]	160	
	Montanez-Heredia et al. [138]	55	
	Ahmad et al. [139]	89	
	Louis et al. [140]	56	
	Filardo et al. [141]	192	Both treatments were effective in improving knee clinical scores. PRP did not demonstrate a clinical superiority compared with HA at any follow-up (up to 6 years, at least)
	Di Martino et al. [142]	192	
PRP/High MWHA /Low MW HA	Kon et al. [143]	150	PRP displayed greater and longer efficacy than HA, as concerning pain, symptom and function improvement; better outcomes were obtained in young and active subjects and lower degree of cartilage damage; worse results were achieved in older patients and more damaged cartilage; in older patients, effects similar to viscosupplementation were obtained
PRP/ PRP+HA/HA	Lana et al. [144]	105	PRP was effective in mild/moderate knee OA; PRP+HA displayed a greater pain and functional limitation decrease than HA alone at 1 year post-injection; increased function compared to PRP alone at 1 and 3 months
PRP/ PRP+HA/HA /normal saline	Yu et al. [145]	360	Combined PRP + HA treatment improved pain, stiffness and physical function compared with PRP or HA alone
PRP/HA /normal saline	Lin et al. [146]	87	Leukocyte-poor PRP provided functional improvement for at least 1 year in mild/moderate OA
PRP/HA/ ozone	Duymus et al. [147]	102	PRP was more effective than HA and ozone
PRP/ PRP+ozone	Dernek et al. [148]	80	Similar efficacy was demonstrated by PRP alone or PRP+ozone; PRP+ozone-treated patients experienced less post-injection pain and a faster recovery
PRP/HA /CS	Huang et al. [149]	120	Pain decrease was significant in all groups compared to baseline; PRP showed a better recovery in physical function and decreasing pain at 6, 9 and 12 months
MP+PRP/ PRP/MP	Camurcu et al. [150]	115	MP+PRP injection determined better clinical improvement compared to PRP and MP alone
PRP double spinning/ PRGF single spinning	Filardo et al. [151]	144	Both treatments displayed similar clinical improvement compared to the baseline and at the follow-up; more pain and swelling reaction were present in PRP patients; younger patients with a low degree of cartilage degeneration showed better results
PRP (6x) + maintenance dose (3x)	Hart et al. [152]	50	PRP decreased pain and improved QoL in low-degree cartilage degeneration. MRI did not confirm cartilage improvement
PRP (1x) /PRP (2x)/ normal saline	Patel et al. [153]	78	Improvement in clinical parameters in both PRP groups; no difference between 1 or 2 injections; results deteriorated after 6 months
PRP (1x) /PRP(3×)/ HA/normal saline	Gormeli et al. [154]	162	PRP and HA treatments are proposed for all OA stages; multiple PRP injections achieved better clinical results in early OA, but did not influence results in advanced OA
PRP large volume	Guillibert et al. [155]	57	Large PRP volume was associated with functional and pain improvement. No MRI difference was reported
PRP+exercise/exercise	Rayegani et al. [156]	62	Short-term improvement of pain, stiffness and QoL in PRP-treated patients compared to the control group was shown
LP-PRP	Duif et al. [157]	58	Improvement of pain and knee function was reported
LP-PRP /saline	Smith et al. [158]	30	Scores in the LP-PRP group were better than in the saline group, starting at 2 weeks throughout
LP-PRP/acetaminophen	Simental-Mendia et al. [159]	65	Better clinical outcomes following LP-PRP treatment were reported
LP-PRP /HA	Cole et al. [160]	99	Similar primary outcomes between HA and PRP were observed at any time point; patient-reported outcome favored PRP; mild OA and low BMI displayed better outcome.
LP-PRP/ HA/NSAID	Buendia- Lopez et al. [161]	106	PRP decreased pain and improved physical function; PRP displayed better results; no modification in cartilage MRI was observed
PRP/ normal saline	Huang et al. [162] Elik et al. [163]	366 60	PRP improved clinical symptoms, improved QoL, decreased joint inflammation and did not increase thickness of cartilage

PRP/SH	Li et al. [164] (Chinese)	30	Significant differences pre- and post-injection in both groups; PRP was better than SH at 6 months
PRP/CS	Forogh et al. [165]	41	Pain, ADL and QoL improvement in the PRP-treated group was greater than in the CS group
PRP/PRL	Rahimzadeh et al. [166]	42	Decreased pain and improved physical function and QoL were observed after both treatments; PRP was more effective
Photo- activated PRP/HA	Paterson et al. [167]	23	Feasibility and safety of PA-PRP treatment were demonstrated; PA-PRP improved pain, symptoms and function; no differences between PA-PRP and HA were observed
PRP+SVF from adipose tissue	Bansal et al. [168]	10	PRP+SVF decreased pain, particularly after 3 months
PRP+intra osseous	Sanchez et al. [169]	14	Knee-joint function improvement and pain decrease were observed in patients with severe OA
PRP+intra-osseous/PRP	Sanchez et al. [170]	60	Intraosseous +intra-articular PRP injections induced better clinical outcome
PRP+intra- osseous/PRP/HA	Su et al. [171]	86	Intra-articular +intraosseous PRP infiltrations were not superior at 2 months, but they were superior at 6 and 12 months

**Table 4:** Evidence of PRP treatment in hip osteoarthritis (reported by year of study and grouped by treatment).

Treatment/ Control	Reference	Patient Number	Main Results
PRP	Sanchez et al. [182]	40	Study supported safety, tolerability and efficacy of PRP treatment; PRR improved pain and function in mild/moderate OA, up to six months
	Singh et al. [183]	36	
PRP/HA	Battaglia et al. [184]	100	PRP showed immediate short-term improvement of pain and function; at 12 months, HA effect was more evident
	Di Sante et al. [185]	43	
	Doria et al. [186]	80	PRP did not display better results than HA in patients with moderate OA
PRP/PRP+ HA/HA	Dallari et al. [187]	111	PRP induced a significant stable pain relief, functional recovery and QoL improvement, up to 12 months; side effects were not observed; improvement was better than PRP+HA or HA alone
PRP+intra-osseous/ PRP	Fitz et al. [188]	Not reported	Intra-articular + intraosseous PRP infiltrations induced improvements at 6 months, but not in the long-term

**Table 5:** Evidence of PRP treatment in Achilles tendinopathy (reported by year of study and grouped by treatment).

Treatment/ Control	Reference	Patient number	Main results
PRP	Gaweda et al. [195]	14	Lasting improvement of the clinical symptoms and imaging results were obtained; improvement was maintained at least for two years from treatment; low complication rate was reported; US-guided tenotomy, followed by PRP treatment, was safe, effective and associated with US improvement; PRP led to tendon matrix healing; effective also in patients who failed to respond to traditional non operative techniques; retrospective study demonstrated that 78% of PRP-injected patients presented clinical improvement and averted surgical intervention at 6-month follow-up; response was less evident in old subjects
	Volpi et al. [196]	15	
	Finoff et al. [197]	41	
	Deans et al. [198]	26	
	Ferrero et al. [199]	30	
	Monto et al. [200]	30	
	Murawski et al. [201]	32	
	Guelfi et al. [202]	73	
	Salini et al. [203]	44	
	Owens et al. [204]	10	Moderate improvement in functional outcome was reported; MRI remained largely unchanged
PRP repeated	Filardo et al. [205]	27	Repeated PRP injections produced overall good outcomes, with stable results up to a midterm follow-up; prolonged symptomatology indicated a difficult return to sport
PRP/normal saline	de Jonge et al. [206]	54	PRP injection in addition to eccentric exercises did not result in clinical and/or ultra-sonographic improvement; tendon diameter increased
	Krogh et al. [207]	24	
LP-PRP/ LR-PRP	Hanisch et al. [208]	84	No significant differences were observed between patients treated with LR-PRP and LP-PRP
PRP+HA	Gentile et al. [209]	10	Treatment was efficacious for tissue healing and regeneration in post-surgical complications of Achilles tendon
PRP+ (ESWT)	Erroi et al. [210]	45	Both PRP and ESWT treatments were similarly efficacious and safe in physically active people
PRP/surgery+ PRP	Oloff et al. [211]	26	Both PRP alone or PRP+ surgical debridement improved clinical outcomes and MRI
PRP/eccentric loading	Kearney et al. [212]	20	No differences between PRP and eccentric loading program as concerning clinical effectiveness
PRP+ eccentric exercise/normal saline+ eccentric exercise	de Vos et al. [213] de Vos et al. [214]	54 54	Patients treated with PRP+ eccentric exercises did not present greater improvement in pain and activity; PRP did not increment tendon structure or modified neovascularization degree
PRP/HVI steroid/ normal saline	Boesen et al. [215]	60	Both HVI steroid or PRP seemed efficacious in improving pain and activity and in decreasing tendon thickness and intra-tendinous vascularity

**Table 6:** Evidence of PRP treatment in lateral epicondyle tendinopathy (reported by year of study and grouped by treatment).

Treatment/ Control	Reference	Patient Number	Main Results
PRP	Mishra et al. [221]	140	PRP was successful in refractory forms and preventing the need for surgery; it was safe and improved function, with effects lasting five years after the initial injection
	Hachtman et al. [222]	31	
	Brkljac et al. [223]	34	
	Brkljac et al. [224]	31	
PRP/Autologous blood	Creaney et al. [225] Thanasas et al. [226]	150 28	PRP seemed to be an effective treatment, superior to autologous blood in short-term, but not in long-term, follow-up; PRP appeared useful in patients resistant to first-line physical therapy

	Raeissadat et al. [227]	75	Both PRP and autologous blood were effective methods; PRP effect was similar to autologous blood
PRP/normal saline	Montalvan et al. [228]	50	PRP treatment was not more effective than saline until 6- and 12-month follow-up
	Schöffl et al. [229]	50	
LR-PRP/LP-PRP	Yerlikaya et al. [230]	90	Neither LR-PRP nor LP-PRP did not seem to affect pain and function in the short-term; leukocyte number was not associated with local inflammation post-injection
PRP/active control	Mishra et al. [231]	230	No differences between treatments were observed at 12 weeks; clinical improvements in PRP-treated patients were observed at 24 weeks
PRP/CS	Peerbooms et al. [232]	100	PRP reduced pain and significantly increased function, exceeding the effect of corticosteroid injection, up to 2 years of follow-up; PRP enabled lesion heling; CS induced a short-term relief at 6 weeks, but favored tendon degeneration
	Gosens et al. [233]	100	
	Gautam et al [234]	30	
	Khaliq et al. [235]	102	
	Varshney et al. [236]	83	
	Gupta et al. [237]	80	
PRP/beta-methasone	Lebiedzinski et al. [238]	120	PRP allowed better results at 12 months; PRP therapeutic effect was long-lasting; betamethasone gave more rapid improvement
PRP/dexa-methasone	Palacio et al. [239]	60	Both treatments were similarly effective
PRP/methyl-prednisolone	Yadav et al. [240]	65	Both PRP and methyl-prednisolone were effective; PRP showed a more prolonged efficacy
PRP/bupivacaine	Behera et al. [241]	25	Leukocyte-poor PRP injection enabled good improvement in pain and function
PRP/laser therapy	Tonk et al. [242]	81	Better results were obtained following PRP injection on the long-term period; low-level laser therapy was better in the short-term
PRP/ESWT	Alessio-Mazzola et al. [243]	63	PRP injection showed a more rapid efficacy than ESWT
PRP/triamcinolone/normal saline	Seetharamaiah et al. [244]	80	Better pain relief were obtained following PRP injection over a short-term period
PRP/glucocorticoids/normal saline	Krogh et al. [245]	60	No treatment was superior to saline in regard to pain reduction; glucocorticoids had a short-term pain-relief effect and reduced both color Doppler activity and tendon thickness, compared with PRP and saline
PRP+dry needling/dry needling	Stenhouse et al. [246]	28	Additional PRP showed a trend to greater clinical improvement in the short-term; no difference between the two treatments was demonstrated at each follow-up
PRP+arthroscopic debridement	Merolla et al. [247]	101	Both PRP injections and arthroscopic debridement were efficacious in short-/medium-term; pain intensified at 2 years in PRP patients; arthroscopic administration favored pain and grip-strength improvement
PRP/ US-guided percutaneous tenotomy	Boden et al. [248]	62	PRP and US-guided percutaneous tenotomy were both successful in improving pain, function and QoL

**Table 7:** Evidence of PRP treatment in plantar fasciitis (reported by year of study and treatment type).

Treatment/ Control	Reference	Patient Number	Main Results
PRP	Ragab et al. [258]	25	PRP injection may have a reparative effect, leading to resolution of symptoms; findings indicated a role in the management of chronic intractable plantar fasciitis; QoL improved; PRP injection was safe; it cannot impair the biomechanical function of the foot; no side effects were reported
	Kumar et al. [259]	44	
	Martinelli et al. [260]	14	
	O’Malley et al. [261]	23	
	Wilson et al. [262]	24	
PRP/PPP	Malahias et al. [263]	36	PRP and PPP gave similar results; both treatments provided improvement at 3- and 6-month follow-up
PRP/normal saline	Johnson-Lynn et al. [264]	28	PRP and placebo gave similar improvement in symptoms
PRP/CS	Aksahin et al. [265]	60	Both treatments were safe and effective in improving pain and function at 3 and 6 months; at 12 months, PRP was significantly more effective, making it better and more durable than CS injection; taking into consideration the potential complication of corticosteroid treatment, PRP injection seemed to be safer and had, at least, the same effectivity in the treatment
	Tiwari et al. [266]	60	
	Omar et al. [267]	30	
	Shetty et al. [268]	60	
	Jain et al. [269]	60	
	Sherpy et al. [270]	50	
	Vahdatpour et al. [271]	32	
	Acosta-Olivo et al. [272]	28	
	Jain et al. [273]	80	
	Monto et al. [274]	40	PRP appeared more effective and durable than CS injection in improving pain and function for the treatment of chronic recalcitrant cases
	Say et al. [275]	50	
	Peerbooms et al. [276]	115	
PRP/methyl-prednisolone	Jiménez-Pérez et al. [277]	40	PRP injection showed better, long-lasting clinical and imaging effects than methylprednisolone
PRP/CS/ normal saline	Mahindra et al. [278]	75	PRP was as effective as, or more effective than, corticosteroid injection at 3-months follow-up
	Shetty et al. [279]	90	PRP and corticosteroids showed superior results to placebo; long-term results and low reinjection and/or surgery rate make PRP more attractive than CS
PRP+ct/ESWT+ct	Chew et al. [280]	54	Either PRP or ESWT treatment resulted in modestly and similarly improved pain and functional scores, compared with conventional treatments alone, over a 6-month follow-up; PRP demonstrated greater improvements in plantar fascia thickness reduction
PRP/DP	Kim et al. [281]	21	Each treatment was effective in chronic recalcitrant cases; PRP also may lead to a better initial improvement compared with DP
PRP/KT	Gonnade et al. [282]	64	PRP injection of high platelet counts was more effective and long-lasting than phonophoresis with kinesiotaping; no adverse effects were reported
PRP/LDR	Gogna et al. [283]	40	PRP and LDR showed similar improvement in pain, functional activity and fascia thickness

**Table 8:** Evidence of PRP treatment in patellar tendinopathy (reported by year of study and treatment type).

Treatment/ Control	Reference	Patient Number	Main Results
PRP	Volpi et al. [196]	15	Significant pain and clinical improvement after 3 months, lasting results up to 2 years; MRI improvement in patellar tendon structure was observed
	Ferrero et al. [199]	28	
	Mautner et al. [288]	27	
	Crescibene et al. [289]	7	
	Kaux et al.[290]	20	
	Bowman et al. [291]	3	Symptoms worsening were described following PRP treatment; poor benefit at 4 months
	Manfreda et al. [292]	17	
PRP (multiple)	Filardo et al. [293]	43	Multiple injections provided good clinical outcomes and stable results, up to medium-term follow-up; patients with bilateral disease and a long history of pain obtained poorer results
PRP(3x)	Charousset et al. [294]	28	Satisfactory results in athletes with chronic tendinopathy and faster return to previous sport practice were reported
PRP(2x)/PRP(1x)	Zayni et al. [295]	40	PRP (2x) determined better results than a single one injection
	Kaux et al. [296]	20	No differences between PRP (2x) and one injection were observed
PRP/Physiotherapy	Filardo et al. [297]	31	PRP treatment significantly improved knee function and quality of life
PRP/PRP+ previous treatment	Gosens et al. [298]	36	PRP provided a significant improvement; no differences were observed between groups
PRP/ESWT	Vetrano et al. [299]	46	PRP led to better midterm clinical results
PRP/HVI image guided saline	Abate et al. [300]	54	Association of both resulted in greater improvement and tendon repair
PRP+dry needling/dry needling	Dragoo et al. [301]	23	PRP provided faster recovery at 12 weeks; no clinical difference at the final 26-week follow-up was observed
LR-PRP/ LP-PRP/ normal saline	Scott et al. [302]	38	LR-PRP or LP-PRP were no more effective than saline for the improvement of symptoms

**Table 9:** Evidence of PRP treatment in muscle injuries (reported by year of study and treatment type).

Treatment/ Control	Reference	Patient Number	Main Results
PRP	Bernuzzi et al. [311]	53	PRP injection under US guide induced a complete muscle-function recovery; pain disappeared; PRP did not accelerate healing but showed excellent muscle repair and small scar
	Zanon et al. [312]	25	
PRP/normal saline	Reurink et al. [313]	80	PRP injection did not demonstrate superiority to normal saline on short-term; no benefits were found up to 12 months in subjective, clinical, MRI measures, return to play and rate of re-injury
	Reurink et al. [314]	80	
	Punduk et al. [315]	12	PRP administration improved inflammatory response induced by high-intensity muscle exercise
PRP/control	Martinez-Zapata et al. [316]	71	PRP did not significantly shorten the time of healing compared to the control group
PRP+conservative treatment/conservative treatment	Bubnov et al. [317] Wetzel et al. [318]	30 15	PRP induced a better physical recovery, decreased pain and promoted faster regeneration than conventional conservative treatment
PRP/CS	Park et al. [319]	56	PRP injection induced more favorable response than CS one week after injection
PRP+rehabilitation/rehabilitation	A Hamid et al. [320] Rossi et al. [321] Borrione et al. [322]	28 75 61	PRP injection+ rehabilitation program induced an earlier full recovery than rehabilitation alone; lower score of pain severity was observed in PRP group; PRP reduced time and costs to reach a complete functional recovery
	Guillodo et al. [323]	34	PRP injection+rehabilitation did not reduce the time to return to play
PRP+rehabilitation/PPP+ rehabilitation/rehabilitation	Hamilton et al. [324]	90	PRP injection+rehabilitation did not show benefit on intensive standardized rehabilitation program alone; PRP induced a more rapid return to sport than PPP

## Abbreviations

ACD	acid citrate dextrose
ADL	activities of daily living
ADP	adenosine diphosphate
A-PRF	advanced-platelet-rich fibrin
ACP	autologous conditioned plasma
BMSCs	bone marrow stem cells
BMP	bone morphogenic protein
CTAD	citrate-theophylline-adenosine-dipyridamole
ct	conventional treatment
CS	Corticosteroid
DP	dextrose prolotherapy
DEPA	dose of platelet efficiency, purity and activation
EGF	epidermal growth factor
EDTA	ethylene diamine tetra-acetic acid
ESWT	extracorporeal shock wave therapy
FG	fibroblast growth factor
GFs	growth factors
HO	heme oxygenase
HS	heparin sodium
HVI	high volume injection
HA	hyaluronic acid
IGF	insulin-like growth factor
ISTH	International Society on Thrombosis and Haemostasis
KT	kinesio therapy
KOOS	knee injury and osteoarthritis outcome score
LE	lateral epicondyle
L-PRF	leukocyte- and platelet-rich fibrin
LP-PRP	leukocyte-poor PRP
L-PRP	leukocyte-rich PRP
LR-PRP	leukocyte-rich PRP
MRI	magnetic resonance imaging
MSC	mesenchimal stem cells
MARSPILL	method, activation, red blood cells, spin, platelets, image guidance, leukocytes and light activation
MIF	migration inhibitory factor
OA	osteoarthritis
PAW	photoactivated
PDWHF	platelet-derived wound healing factors or formula-
PF	plantar fasciopathy
PRFM	platelet-rich fibrin matrix
PRGF	plasma rich in growth factors
PAW	platelet activation, white blood cells
PCs	platelet concentrates
PLRA	platelet leukocyte red blood cells and activation
PRP	platelet rich-plasma
PDGF	platelet-derived growth factor
PPP	platelet-poor plasma
PRF	platelet-rich fibrin
P-PRF	pure platelet-rich fibrin
QoL	quality of life
RBCs	red blood cells
SC	sodium citrate
t-PRP	temperature controlled PRP
TGF	transforming growth factor
US	ultra-sound
VEGF	vascular endothelial growth factor
VAS	visual analogue scale
WOMAC	the Western Ontario and McMaster Universities Osteoarthritis Index
